# Preparation of Co-Processed Excipients for Controlled-Release of Drugs Assembled with Solid Lipid Nanoparticles and Direct Compression Materials

**DOI:** 10.3390/molecules26072093

**Published:** 2021-04-06

**Authors:** Luis Eduardo Serrano-Mora, María L. Zambrano-Zaragoza, Néstor Mendoza-Muñoz, Gerardo Leyva-Gómez, Zaida Urbán-Morlán, David Quintanar-Guerrero

**Affiliations:** 1 Laboratorio de Investigación y Posgrado en Tecnología Farmacéutica, Universidad Nacional Autónoma de México, FES-Cuautitlán, Cuautitlán Izcalli 54745, Estado de México, Mexico; luedserrano_h@hotmail.com; 2 Laboratorio de Procesos de Transformación y Tecnologías Emergentes de Alimentos, Universidad Nacional Autónoma de México, FES-Cuautitlán, Cuautitlán Izcalli 54714, Estado de México, Mexico; luz.zambrano@unam.mx; 3 Laboratorio de Farmacia, Facultad de Ciencias Químicas, Universidad de Colima, Colima 28400, Mexico; nmendoza0@ucol.mx; 4 Departamento de Farmacia, Facultad de Química, Ciudad Universitaria, Universidad Nacional Autónoma de México, Circuito Exterior S/N, Del. Coyoacán, Ciudad de México 04510, Mexico; gerardoleyva@hotmail.com; 5 Laboratorio de Cromatografía, Facultad de Química, Universidad Autónoma de Yucatán, Mérida 97069, Yucatán, Mexico; zaida.urban@correo.uady.mx

**Keywords:** dicalcium phosphate dihydrate, solid lipid nanoparticles, co-processed excipient, ranitidine hydrochloride, controlled release

## Abstract

The purpose of the study was to develop a novel, directly compressible, co-processed excipient capable of providing a controlled-release drug system for the pharmaceutical industry. A co-processed powder was formed by adsorption of solid lipid nanoparticles (SLN) as a controlled-release film onto a functional excipient, in this case, dicalcium phosphate dihydrate (DPD), for direct compression (Di-Tab^®^). The co-processed excipient has advantages: easy to implement; solvent-free; industrial scaling-up; good rheological and compressibility properties; and the capability to form an inert platform. Six different batches of Di-Tab^®^:SLN weight ratios were prepared (4:0.6, 3:0.6, 2:0.6, 1:0.6, 0.5:0.6, and 0.25:0.6). BCS class III ranitidine hydrochloride was selected as a drug model to evaluate the mixture’s controlled-release capabilities. The co-processed excipients were characterized in terms of powder rheology and dissolution rate. The best Di-Tab^®^:SLN ratio proved to be 2:0.6, as it showed high functionality with good flow and compressibility properties (Carr Index = 16 ± 1, Hausner Index = 1.19 ± 0.04). This ratio could control release for up to 8 h, so it fits the ideal profile calculated based on biopharmaceutical data. The compressed systems obtained using this powder mixture behave as a matrix platform in which Fickian diffusion governs the release. The Higuchi model can explain their behavior.

## 1. Introduction

The preference for manufacturing tablets as a controlled-release drug system can be enhanced by incorporating new materials into the compression process. The direct-compression process’s simplicity and cost-effectiveness have positioned it as an alternative to the traditional granulation technology [1,2,3]. The success of this process is critically affected by the powder’s behavior in terms of flowability and compressibility [4]. Formulations of the kind explored in the present study contain an excipient at a higher concentration than the active pharmaceutical ingredient (API), so it significantly influences the processing, stability, safety, and performance of this form of solid dosage [5]. Most currently available excipients fail to meet the desired set of functional criteria, so there is a need to create high-functionality excipients (HFI) [6]. HFI can be obtained through various means: developing new chemical formulations, enhancing existing ones, or elaborating new combinations of existing excipients [7]. Developing new chemical excipients entails significant economic and operational challenges related to safety and regulatory norms [8].

Developing excipients with improved physico-mechanical properties is drawing the attention of pharmaceutical companies as they seek new formulations to enhance their products [9]. No single excipient possesses all the desired physico-mechanical properties for developing a robust system [10,11]. There is a need for excipients that possess specific critical characteristics, such as better flow rates, low or null sensitivity to moisture, superior compressibility, and the capacity for fast disintegration [12]. To date, the development of such excipients has been market-driven (that is, first they are developed and then market demand is created through commercialization strategies), but activity in this field has been minimal, as shown by the fact that no new chemical excipient has been introduced into the market in recent years [10,13].

Co-processing is one of the most widely-explored and commercially-utilized methods for developing new excipients and the resulting products that combine two or more excipients to obtain performance advantages superior to simple physical mixtures [9,10]. This process involves the following steps: selecting the excipients required, determining their optimal relative proportions, choosing the most suitable co-processing technique, and optimizing various process parameters. Most co-processed excipients combine a filler with a binder, a binder with a glidant, or a filler with a binder and a glidant [14,15]. 

The availability of nanotechnology opens opportunities to elaborate on new co-processed excipients. Solid lipid nanoparticle (SLN) technology is emerging as an option for developing alternative colloidal carrier systems for controlled and targeted delivery [16]. Nanoparticles are of submicron size (50–1000 nm). Common ingredients used in their formulation include lipids (matrix materials), emulsifiers, co-emulsifiers, and water [16]. These carriers are composed of physiological and biodegradable lipids of low systemic toxicity and cytotoxicity [17,18]. Glyceryl behenate (Compritol^®^ 888 ATO; Gattefossé, St Priest, Cedex, France) is one agent used to formulate controlled-release tablets [17,19,20,21,22,23].

To our knowledge, there is not available in the literature or commercially a co-processed system assembled by SLN on a compressible insoluble powder. The present study aimed to formulate and then characterize a novel controlled-release excipient for pharmaceutical applications using a simple method based on the adsorption of SLN onto a direct, insoluble compression powder. 

The system was evaluated to determine its rheological and compressibility properties and its capacity to function as a drug-delivery platform for a drug model’s controlled release (ranitidine hydrochloride).

## 2. Results and Discussion

### 2.1. Particle Size of the SLN

The SLNs of Compritol^®^ 888 ATO were prepared efficiently by the hot dispersion method. The particle size obtained was in the range of 269 ± 6 nm. The polydispersity index (PDI) was narrow for all the batches with values of 0.29 ± 0.05, indicating a homogeneous particle size distribution. These results show a smaller particle size and PDI than those reported by Martinez-Acevedo et al. [24] for Compritol^®^ 888 ATO SLN using polyvinyl alcohol as the stabilizer and the hot dispersion method, and are consistent with those reported by Swapnil-Kumar et al. [25] for Compritol^®^ 888 ATO SLN loaded with ritonavir and prepared by the solvent evaporation method.

### 2.2. Particle Size Analysis of the Co-Processed Excipients

The Di-Tab^®^ (Rhodia, Inc., Cranbury, NJ, USA):SLN ratio that produced the best co-processed excipient was selected based on the rheological properties of the material and the capacity to form high-quality compressed tablets. The results are summarized in Table 1. The smallest particle size, 92.7 ± 9.7 µm, occurred in batch 1 with a Di-Tab^®^:SLN ratio of 4:0.6. Similar results were obtained for batch 2, at 96.9 ± 1.5 µm with a Di-Tab^®^:SLN ratio of 0.5:0.6 [26]. Batches 3 and 4 had Di-Tab^®^:SLN ratios of 2:0.6 and 1:0.6, respectively, and particles size of 105.8 ± 4.8 and 114.7 ± 3.4 µm, respectively, like those of Di-Tab^®^ as a raw material. These results suggest that it is possible to control particle size through the granulation step that produces a non-binding powder in which the lipidic layer does not show a cohesive behavior, at least not at these concentrations.

In contrast, batches 5 and 6, with Di-Tab^®^:SLN ratios of 0.5:0.6 and 0.25:0.6, showed particles sizes of 205 ± 2.3 and 208.8 ± 2.5 µm, respectively, almost two-fold greater than the particle size of Di-Tab^®^. It seems clear that an excess of Compritol^®^ 888 ATO plays an essential role in the size and shape of the particles by affecting aggregation and/or strong binding among them. This section may be divided by subheadings. It should provide a concise and precise description of the experimental results and their interpretation, as well as the experimental conclusions that can be drawn.

### 2.3. Microscopy Electron Scanning

**Figure 1:**
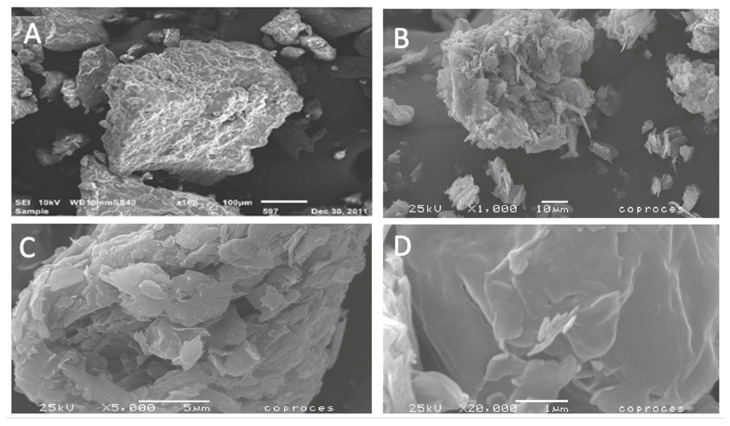
SEM photographs of (**A**) Di-Tab, (**B**) batch 3 at magnification of 1000X (**C**) batch 3 at magnification of 5000X, and (**D**) batch 3 at magnification of 20,000X.

Figure 1 shows the scanning electronic micrographs of Di-Tab^®^ and the co-processed batch (batch 3; Di-Tab^®^:SLN ratio of 2:0.6). Pure Di-Tab^®^ had a spherical morphology with high porosity, revealing monoclinic crystals, which was characteristic of its preparation method. The co-processed surface particles appeared smoother than that of Di-Tab^®^, suggesting the formation of a thin film by the adsorption and coalescence of SLN onto the surface of Di-Tab^®^.

### 2.4. Rheology Powders of the Co-Processed Excipients

Table 1 shows the rheological properties of the co-processed excipients, indicating that the physical characterization of the powders plays a crucial role in the pharmaceutical manufacturing process [27]. Batches 5 and 6, with Di-Tab^®^:SLN ratios of 0.5:0.6 and 0.25:0.6, respectively, had the highest values for both densities, while batch 1 (Di-Tab^®^:SLN ratio 4:0.6) presented the lowest values for the bulk and tap densities, of just 0.53 and 0.73, respectively. These results show a tendency related to the Di-Tab^®^:SLN ratio in which high concentrations of Di-Tab^®^ resulted in low values for both densities. The bulk and tap densities were used to determine the Carr and Hausner indexes. The usefulness of these two indexes lies in their ability to predict the flow capacity of a powder [28]. Particle size and shape and cohesive force all exhibited relations to the Carr and Hausner indexes. Batches 5 and 6, with Di-Tab^®^:SNL ratios of 0.5:0.6 and 0.25:0.6, respectively, had the highest values. The results obtained for the Hausner index depicted values of 1.19–1.22 for all powders, suggesting that they had adequate flow capacities. Meanwhile, the Carr index values revealed adequate flow capacities for all the co-processed excipients with values between 16 and 20 [29,30,31].

The angle of repose is a relative measurement of the friction and cohesion among particles, so the particle size of the material will influence its flow capacity [32,33]. Our results demonstrated a tendency towards higher values for the angle of repose as the amount of Di-Tab^®^ added to the batch decreased. The lowest values for this parameter occurred in batches 1 and 2 with Di-Tab^®^:SLN ratios of 4:0.6 and 3:0.6, respectively, at values of 24 ± 2.5°. Batches 5 and 6, which had lower proportions of Di-Tab^®^ and Di-Tab^®^:SLN ratios of 0.50:0.6 and 0.25:0.6, respectively, showed higher values for the angle of repose of 28 ± 2.1° and 32 ± 4.2°. This tendency was related to the amount of Compritol^®^ 888ATO that was adsorbed onto the surface of the Di-Tab^®^. A low proportion of Di-Tab^®^ favored the cohesive force on the material, forming larger, more irregularly shaped particles that presented more significant interparticle interaction and a higher angle of repose in batches 5 and 6. According to the values established in the USP42-NF 37, all the batches of co-processed excipients showed excellent flow with values of 24–32°. This suggests that these materials can withstand the direct compression process [31,34].

The flow rate of a material depends on the relation between the particles and the process [3,9]. The flow rate of a powder is a property that indicates the friction among the constituent particles [31,35]; hence, particle size and morphology are determinant factors for the flow rate of any material. Table 1 shows the results of the determination of the flow rate. The Di-Tab^®^:SLN ratios of 2:0.6 and 1:0.6 presented the highest free flow rates, with values of 3.67 ± 0.56 and 3.80 ± 0.62 g/s, respectively. These batches were the ones that had a particle size similar to Di-Tab^®^, which generated a flow rate of 10.82 ± 0.60 g/s. The Di-Tab^®^:SLN ratios of 4:0.6, 3:0.6, 0.5:0.6, and 0.25:0.6 presented similar values that ranged from 2.34 ± 0.21 to 3.00 ± 0.34 g/s. These data suggest the effects of particle size and the amount of Di-Tab^®^ on the flow rate. For the batches with lower amounts of Di-Tab^®^, the interparticle cohesive forces induced by a higher proportion of SLN Compritol^®^ 888 ATO showed a negative effect on the flow rate. These mixtures had particle sizes that were either larger or smaller than that of Di-Tab^®^, so they modified the morphology of the particles and produced more significant intraparticle interaction that affected the free flow of the excipients.

### 2.5. Packing and Cohesive Properties

**Figure 2:**
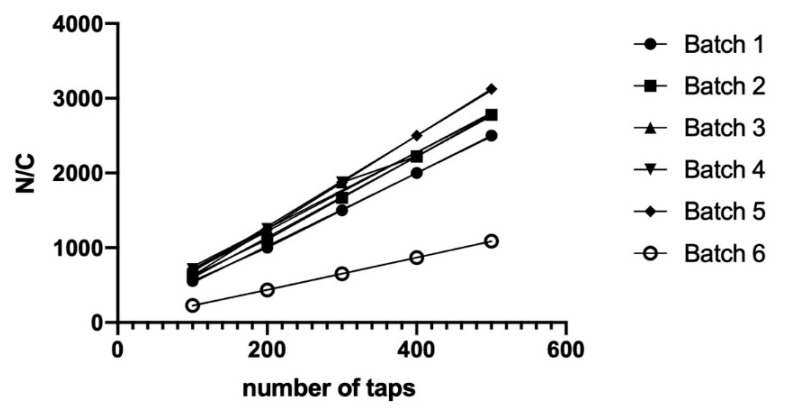
Behavior of co-processed excipients during packing N/C vs. number of taps.

Particle size, particle shape, particle size distribution, and the intra- and interparticle size play a complex role in the flow properties. The Kawakita equation’s constants were resolved from the plots of N/C against the number of taps, as shown in Figure 2. 

The Kawakita’s constants a and b for each of the excipients evaluated are listed in Table 2. In terms of the “a” constant, a higher value is related to poor flowability, being batch 6, with the highest compressibility value of 0.464, exhibited the lowest flowability. The other batches showed “a” value between 0.165 and 0.205, batch 1 presented the highest “a” value. The cohesive force was related to the constant 1/b, where lower values showed less cohesive force. The most cohesive excipients were batches 3 and 4. The results obtained by Kawakita analysis are consistent with the rheologic characterization by Carr and Hausner index.

The value for the constant “a” was related to the shape and particle size. Spherical particles presented an “a” value smaller than those observed for irregular particles. On the other side, smaller particle sizes increased the “a” values [36]. According to the results for packing co-processed prepared behavior, the highest “a” value was for batch 6, suggesting that the shape played a significant role in its particle size.

### 2.6. Evaluation of the Compressibility Behavior of the Co-Processed Excipients

The co-processed excipients’ consolidation behavior was determined from the results of a tensile strength test, which were adjusted to the criteria established.

The results obtained by ANOVA analysis on the tensile strength test were analyzed by Student’s *t*-test (*p* > 0.05) and are shown in Table 3. The co-processed excipient obtained showed no influence of mixing consolidation times; these results are consistent for materials that present fragmentable behavior. Various authors [26,37,38] have described the brittle behavior of compacted dibasic calcium phosphate dihydrate. Almaya et al. [37], for instance, determined that viscoelastic and deformed plastic materials were more sensitive to the addition of a lubricant than brittle materials, suggesting that tensile strength was independent of the addition of a lubricant and of the lubricant mixing time. The comportment is a characteristic of a material whose primary deformation mechanism is a brittle fracture. Ideal diluents should comprise a mixture of a component that fragments—i.e., brittle—and one that deforms a plastic, as this will incorporate the advantages of both mechanisms [3]. The co-processed compressibility behavior was not affected by the assembling process at all proportions tested.

### 2.7. Dissolution Studies

**Figure 3:**
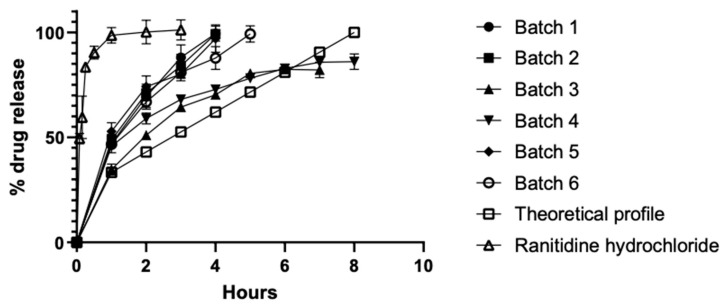
Drug release profile of ranitidine hydrochloride from different co-processed excipients.

Figure 3 shows the in vitro drug release profiles of the six co-processed batches compared to the ranitidine hydrochloride (control) and the theoretical controlled-release profile. The oral controlled release of ranitidine hydrochloride was estimated based on its pharmacokinetic parameters using Equation (1), where MD is the maintenance dose and IR is the immediate release dose, which depends on the concentration steady state, the distribution volume, the bioavailable fraction, and t_1/2_, which is the half-life [39]. The ranitidine hydrochloride tablet showed a fast dissolution, reaching 100% of the drug released within the first hour. The best correlation with the theoretical profile was found for batches 3 and 4 with Di-Tab^®^:SLN ratios of 2:0.6 and 1:0.6, respectively. Batches 1, 2, 5, and 6 presented fast release, as 80% of the drug was released within 1–4 h.

(1) $$DM=IR\;\times \;\left(\frac{1+ln2t}{t_{\frac{1}{2}}}\right)$$

**Figure 4:**
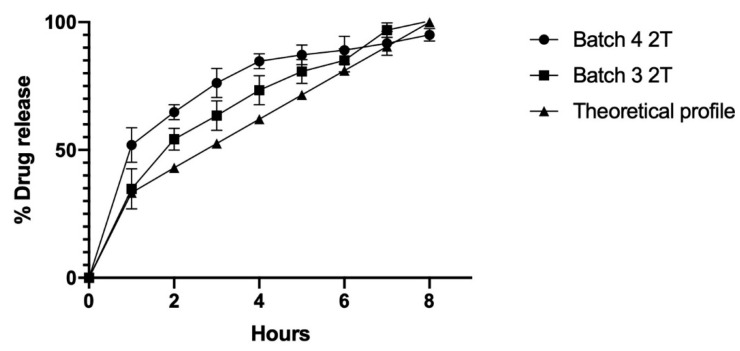
Drug release profile of ranitidine hydrochloride from co-processed excipient batches 3 and 4 at 2 tons of compression force.

An analysis that compared each batch dissolution profile to the theoretical profile was performed using the similarity factor (f2), as shown in Table 4. The best approach was observed with batches 3 and 4, which had f2 values of 22.13 and 21.71, respectively. These batches were then examined in the additional optimization step in order to fit them into the release profile. For this step, new batches (3 and 4) were prepared using a compression force of 2 tons to reduce the release rate, since a greater compression force can decrease interparticle porosity [40], slow wetting, and lower the release speed. The results show that batch 3—Di-Tab^®^:SLN ratio of 2:0.6—had the best fit, with a similarity factor of 54.64 (optimized batch) as it is observed in Figure 4. In this regard, Trivedi et al. determined a correlation that was even better than the theoretical profile for a floating matrix tablet of ranitidine, by achieving an f2 value of 69.5.

Various release models (semi-empirical Korsmeyer–Peppas, Higuchi, zero-order, and first-order) were tested to fit the release profiles of the formulations developed. The Korsmeyer–Peppas approach is a semi-empirical model that makes it possible to infer the type and mechanism of drug release in both matrix and membrane drug-delivery systems [41]:

(2) $$\frac{M_{t}}{M_{\infty }}=kt^{n}$$

*M_t_* is the amount released at time *t*, *M_α_* is the maximum amount released, *k* is a dimension that includes the macromolecular system’s properties, and *n* is an exponential that is indicative of the drug release type and release mechanism. Thus, a value of *n* = 0.5 describes a Fickian diffusion release mechanism (case I, time-dependent t^0.5^), where values to the order of 0.5 < *n* < 1.0 reflect an anomalous, or non-Fickian, behavior (time-dependent t^0.5^ to t^1.0^), and a value of *n* = 1 shows a zero-order kinetic mechanism (case II, time-independent) [10,42,43,44,45]. Upon applying the Korsmeyer–Peppas model, a high correlation coefficient (r^2^ = 0.9919) was found for the optimized batch 3, Di-Tab^®^:SLN ratio of 2:0.6 (Table 5). The exponential “*n*” value was 0.5176, suggesting a Fickian diffusion release (time-dependent, t^0.5176^), where diffusion of the ranitidine hydrochloride was due to the potential chemical gradient. These results are consistent with the Higuchi model (r^2^ 0.9892), which establishes a K_H_ value of 32.98. These results show that the co-processed excipients elaborated in this study form compact platforms by direct compression, in which the SLN plays an essential role in releasing the drug encapsulated in the matrix system. 

## 3. Materials and Methods

### 3.1. Materials

The direct compression process of materials (dicalcium phosphate dihydrate, Di-Tab^®^; Innophonos) and the drug ranitidine hydrochloride (˃99%) were donated by HELM Mexico. Glyceryl behenate (Compritol^®^ 888 ATO; Gattefossé) was purchased from Lyontec (Mexico City, Mexico). The stabilizing agent, Pluronic F-68^®^, was obtained from Aldrich (Merck Chemicals GmbH, Germany). Distilled water was of Milli-Q quality (Millipore, Bedford, MD, USA). All other reagents were of at least analytical grade.

### 3.2. General Description

**Figure 5:**
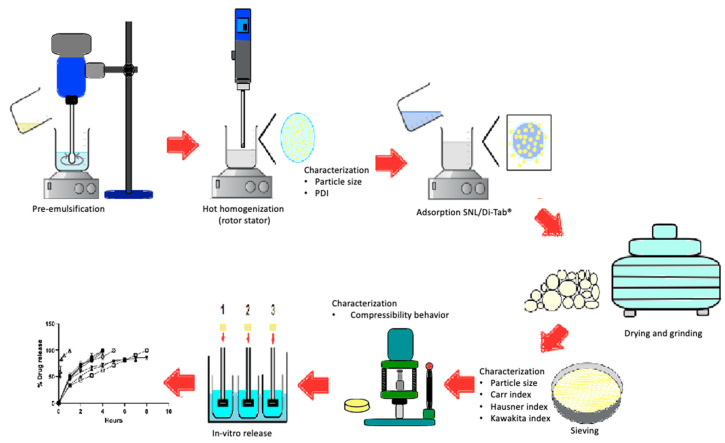
Experimental procedure to obtain a co-processed excipient.

Figure 5 summarizes the experimental design to prepare the co-processed excipient from the SLN of Compritol^®^ 888 ATO and to direct the compression excipient and its evaluations during the different process steps. 

### 3.3. Preparation of Solid Nanoparticles of Compritol^®^ 888 ATO

The SLN of Compritol^®^ 888 ATO were obtained using the hot high-energy (rotor-stator) dispersion method. This process is one of the most used because of its avoidance of the use of solvents, its simple implementation, and its easy scaling-up. Briefly, 8 g of lipid (internal phase) was melted at 5 degrees above the material’s melting point (85 °C). The lipid was then dispersed in 92 mL of an aqueous solution containing 2.5% (*w*/*w*) Pluronic^®^ F-68 as a stabilizer at the same temperature used to melt the lipid. A pre-emulsion was then formed by mechanical stirring for 5 min at 1200 rpm. The oil/water emulsion obtained was homogenized in a high-efficiency disperser (rotor-stator system, Ultra-Turrax^®^ apparatus, T18 IKA; Labortechnik, Wilmington, DE, USA) at 10,000 rpm for 5 cycles of 5 min each with a 3 min repose period between cycles. The number of cycles and dispersion time were selected from a previous work [24]. The system was allowed to cool to room temperature.

### 3.4. Particle Size Analysis

The laser light-scattering technique determined the average particle size and polydispersity index (Coulter^®^ N4, Coulter, Florida, USA). Measurements were obtained at a 90° fixed angle for 180 s at 25 °C. The dispersions were diluted with water, and measurements were made in triplicate for all batches prepared.

### 3.5. Preparation of the Co-Processed Excipients Based on SLN Compritol^®^ 888 ATO/Di-Tab^®^

The co-processed excipients were prepared for adsorption of the SLN of Compritol^®^ 888 ATO on Di-Tab^®^. Six systems with different Di-Tab^®^:SLN ratios were prepared, as summarized in Table 6. Initially, 750 mL of the 8% w/w dispersion of SLN and 250 mL of water were placed in a glass container. Di-Tab^®^ was added slowly under mechanical propeller stirring (IKA^®^, RW 28 basic) at 1200 rpm for 24 h. Finally, the systems were dried in an air oven (NESCO FD-75PR 5, Milwaukee, USA) at 45°C. Subsequently, the mass was granulated using the fraction that passed through a 100-mesh sieve (less than 149 µm).

### 3.6. Particle Size Analysis of the Co-Processed Excipients

The average particle size of the co-processed excipients was determined by laser diffraction (Malvern 2000, Malvern Instruments Ltd., England) based on Fraunhofer’s approach. The powders were dispersed in water, and measurements were made in triplicate for all batches prepared.

### 3.7. Microscopy Electron Scanning

A drop of concentrated co-processed aqueous dispersion was placed in a sample holder to apply a gold bath (~20 nm) using a cathode evaporator (Sputter Coater JFC-1100 Jeol, Japan). The samples were observed in a LV-SEM JSM 5600 low-vacuum scanning electron microscope (5 nm resolution), with a voltage of 20 kV and 12–20 Pa of pressure in the chamber.

### 3.8. Rheology of the Powders

#### 3.8.1. Angle of Repose

Each powder’s angle of repose was measured using the fixed funnel method. The funnel height was adjusted to the bottom edge and maintained at 10 cm from the test surface. The cone angle to the horizontal plane was recorded. The tangent to the angle of repose was the coefficient of internal friction. The relation between flow properties and the angle of repose was established in USP42-NF 37, determined according to Equation (3) [46].

(3) $$\theta ={tan}^{-1}\left(\frac{h}{r}\right)$$

#### 3.8.2. Density, Carr, and Hausner Indexes

The bulk and tap densities of the excipients were determined using the following method. A 50 mL glass cylinder was weighed, filled with 30 g of powder, and then reweighed. Bulk density was registered by the volume that the co-processed excipient occupied before mechanical tapping. The tap volume was measured after 100, 200, 400, and 500 taps at the height of 14 mm in a tap density tester (model SWM 22, Erweka, Heusenstamm, Germany). Each analysis was repeated three times.

The Carr and Hausner indexes were determined based on the values that corresponded to the bulk (*d_bulk_*) and tap (*d_tap_*) densities. These were measured after administering 100 taps to a sample of the co-processed excipient. The indexes were calculated with the following equations:

(4) $$Carr\;index\;\left(CI\right)=\left(\frac{\left(d_{tap}-d_{bulk}\right)}{d_{tap}}\right)$$

(5) $$Hausner\;index\;\left(CH\right)=\left(\frac{d_{tap}}{d_{bulk}}\right)*100$$

The flow capability of each powder was analyzed based on the criteria reported in USP42-NF 37 [46].

#### 3.8.3. Determination of Packing and Cohesive Properties

The packability was determined using the Kawakita analysis which was developed to study powder densification using the degree of reduction in volume. The Kawakita equation is given by:

(6) $$\frac{N}{C}=\frac{1}{ab}+\frac{N}{a}$$

where “*a*” represents the proportion of consolidation at closets packing (compactibility) prior to compression, and “1/b”, also known as the coefficient of compression, is related to cohesion. *C* is the degree of volume reduction, calculated from the initial volume *Vo* and tapped volume *Vn* as

(7) $$C=\frac{Vo-Vn}{Vo}$$

where *Vo* is the initial volume of the powder bed and Vn is the change in volume of the powder bed noted after tap number. The numerical values for constants a and 1/b were obtained from the slope of the plot of N/C versus the number of taps, N (N = 100, 200, 300, 400, and 500).

### 3.9. Evaluation of the Compressibility Behavior of the Co-Processed Excipients

This determination was performed following the procedure described in Wells [47], which consisted of preparing three batches (A, B, and C) of tablets (*n* = 6) using a single-punch, manual tablet machine (Carver Laboratory Press, Perkin Elmer, USA). The flat punch had a diameter of 1.13 cm and a compression force of 1 ton. Each compressed tablet contained 750 mg of one of the co-processed excipients and 7.5 mg of magnesium stearate. The process variables analyzed were consolidation time and mixing. The first batch (A) was mixed for 5 min and compacted for 1 s; the second (B) was mixed for 5 min and compacted for 30 s; and the third (C) was mixed for 30 min and compacted for 30 s. The tensile strength (TS) of each kind of compressed tablet (*n* = 3) was measured using a hardness tester (Pharma Test type PBT 301). Data were analyzed by comparing the average TS of batches A, B, and C, as shown in Table 7, allowing us to infer the different powders’ behavior during compression [47].

### 3.10. Dissolution Studies

Ranitidine hydrochloride—class III of the biopharmaceutics classification—was used as the model drug because it has a low oral bioavailability of 50–60%, a high distribution volume, and a half-life of 2–3 h, making it the right candidate for controlled-release oral administration [48]. The tablets (700 mg) were prepared using 1.13 mm concave punches, compressed in the aforementioned manual tablet machine under a force of 1 ton, and allowed a consolidation time of 15 s. The co-processed tablets were made from a powder mix containing 336 mg of ranitidine hydrochloride and 364 mg of one co-processed excipient. The control tablets had an equivalent amount of ranitidine hydrochloride and Di-Tab^®^ as filler.

Dissolution studies (*n* = 6) were conducted using the USP 42-NF 37 paddle apparatus (optimal control mod. DT1, USA). The compressed tablets were placed in a dissolution medium (900 mL of 0.1 N HCl) and maintained at a temperature of 37 ± 1 °C. The stirring speed was 75 rpm. Aliquots measuring 10 mL were taken at several time intervals (1, 2, 3, 4, 5, 6, 7, and 8 h) and filtered using a 0.22 µm Millipore syringe filter. In order to maintain a constant initial volume, an equivalent volume of dissolution medium was replaced. The concentration of ranitidine hydrochloride in the filtered aliquots was measured by UV spectrophotometry (Varian Cary^®^ 50 Scan UV Visible Spectrophotometer) at 313 nm; the corresponding concentrations of the drug were calculated through the calibration curve. This analytical method was validated over a concentration range of 16–96 µg/mL. Results are reported as means ± standard deviations for three independent tests, as each test was performed in triplicate. The drug release profiles for all formulations were compared to the theoretical controlled-release profile for eight hours reported by Travedi et al. [39]. All formulations of the compacted ranitidine hydrochloride were analyzed using least-squares linear regression, fitting the dissolution data into different release models to obtain the rate and release mechanism.

## 4. Conclusions

This study developed a series of novel, versatile, and straightforward controlled-release co-processed excipients assembled with nanoparticles and a direct compressed pharmaceutic excipient. The method proposed to prepare these co-processed excipients had different advantages, such as (i) easy to implement; (ii) solvent-free; (iii) possible industrial scaling-up; (iv) good rheological and compressibility properties; and (v) capability to form inert compacts that work as a release platform. The best Di-Tab^®^:SLN ratio was determined to be 2:0.6 for batch 3, which had good flow properties and the compatibility necessary to withstand the direct compression process. These co-processed systems could control the release rate for an extended time by an easy manufacturing process by direct compression. It was possible, as well, to develop a similar controlled-release system for ranitidine chloride—8 h—by modifying the compression force. Other means of controlling release—e.g., adding soluble fillers—are currently being studied. The behavior of the co-processed excipients fit the Higuchi model for matrix systems. In conclusion, this optimized co-processed excipient could provide a tool that will help resolve the lack of new, functional excipients for the pharmaceutics industry that are free of chemical changes and enhance the properties of the raw excipients. 

## Figures and Tables

**Table 1:** Rheological properties and particle size obtained for the co-processed excipients (NLS Compritol^®^ 888 ATO/ Di-Tab^®^) assembled.

System	Particle Size (µm)	Bulk Density	Tap Density	Carr Index	Hausner Index	Angle of Repose	Flow Rate (g/s)
Di-Tab^®^	128.5	0.83 ± 0.20	0.97 ± 0.23	14 ± 1	1.13 ± 0.06	30.0° ± 1.8	10.82 ± 0.60
Compritol^®^ 888 ATO	nd *	0.36 ± 0.11	0.49 ± 0.09	26 ± 2	1.34 ± 0.08	62.0° ± 3.3	1.26 ± 0.40
Batch 1	92.7 ± 4.9	0.53 ± 0.12	0.73 ± 0.11	16 ± 1	1.19 ± 0.04	24.0° ± 2.5	2.34 ± 0.21
Batch 2	96.9 ± 1.5	0.64 ± 0.15	0.73 ± 0.12	16 ± 1	1.19 ± 0.05	24.0° ± 1.8	3.00 ± 0.34
Batch 3	114.7 ± 3.5	0.61 ± 0.15	0.73 ± 0.11	16 ± 1	1.19 ± 0.04	21.0° ± 2.7	3.67 ± 0.56
Batch 4	105.8 ± 4.8	0.65 ± 0.10	0.77 ± 0.11	16 ± 1	1.19 ± 0.03	26.0° ± 2.3	3.80 ± 0.62
Batch 5	208.0 ± 2.5	0.72 ± 0.18	0.88 ± 0.13	18 ± 1	1.22 ± 0.05	28.0° ± 2.1	3.05 ± 0.30
Batch 6	205 ± 2.3	0.71 ± 0.12	0.87 ± 0.13	20 ± 1	1.22 ± 0.03	32.0° ± 4.2	3.33 ± 0.72

* nd: not determined.

**Table 2:** Kawakita’s constants.

Batch Number	a	1/b
Batch 1	0.205	9.09
Batch 2	0.185	10.26
Batch 3	0.189	31.58
Batch 4	0.196	46.69
Batch 5	0.165	11.76
Batch 6	0.464	3.67

**Table 3:** Student’s *t*-test to determine the compressibility behavior of the assembled co-processed excipients.

Batch	A = B				A = C			
	Mean		t-Statistic	t-Critical	Mean		t-Statistic	t-Critical
	A	B			A	C		
1	10.27	13.27	−3.19	2.78	10.27	11.80	−2.64	2.78
2	12.20	12.43	−0.22	2.78	12.20	11.80	0.64	2.78
3	12.10	13.27	−1.10	2.78	12.10	11.90	0.39	2.78
4	10.63	11.07	−0.98	2.78	10.63	10.87	−0.48	2.78
5	7.17	7.33	−0.61	2.78	7.17	7.33	−0.61	2.78
6	13.13	12.70	1.07	2.78	13.13	13.00	0.39	2.78

**Table 4:** Similarity factor (f2) of the dissolution profile of ranitidine chloride for different co-processed systems.

Batch	Similarity Factor
1	9.48
2	9.53
3	22.13
4	21.71
5	9.55
6	11.51
4 *	38.83
3 *	54.64

* at 2 tons of compression force.

**Table 5:** Correlation coefficient (r^2^) for different release models.

Batch Number	Zero-Order	First-Order	Higuchi Model	Korsmeyer–Peppas Model	
				r^2^	n
1	0.9288	0.9458	0.9973	nd *	nd *
2	0.9164	0.9529	0.9969	nd *	nd *
3	0.8407	0.8250	0.9931	0.9945	0.5177
4	0.7488	0.8571	0.9876	0.9951	0.3327
5	0.8711	0.9363	0.9694	nd *	nd *
6	0.8827	0.9168	0.9917	nd *	nd *
4 2T	0.7180	0.8243	0.9916	0.9974	0.3535
3 2T	0.9051	0.8901	0.9892	0.9899	0.5176

* nd: not determined.

**Table 6:** Compritol^®^ 888 ATO SLN/ Di-Tab^®^ ratios for the co-processed excipients assembled.

Batch	Di-Tab^®^ (g)	SLN 8% (*w*/*v*) of Compritol^®^ 888 ATO (mL)	Distillate H_2_O (mL)	Ratio Di-Tab^®^:SLN
1	400	750	250	4:0.6
2	300	750	250	3:0.6
3	200	750	250	2:0.6
4	100	750	250	1:0.6
5	50	750	250	0.5:0.6
6	25	750	250	0.25:0.6

**Table 7:** Compressibility behavior of excipients from tensile strength determinations according to Wells [47].

Average Tensile Strength	
**Plastic**	**Brittle**
A<B	A=B
A>C	A=C
C<A<B	A=B=C

## Data Availability

The data presented in this study are available on request from the corresponding author.

## Sample Availability

Not available.
